# Medicinal Uses of the Fabaceae Family in Zimbabwe: A Review

**DOI:** 10.3390/plants12061255

**Published:** 2023-03-10

**Authors:** Alfred Maroyi

**Affiliations:** Department of Botany, University of Fort Hare, Private Bag X1314, Alice 5700, South Africa; amaroyi@ufh.ac.za

**Keywords:** Fabaceae, ethnobotany, herbal medicine, Leguminosae, natural compounds, traditional knowledge, Zimbabwe

## Abstract

The current study is aimed at providing a systematic review of the ethnomedicinal, phytochemical and pharmacological properties of Fabaceae species used as sources of traditional medicinies in Zimbabwe. Fabaceae is one of the well-known plant families of ethnopharmacological importance. Of the approximately 665 species of the Fabaceae family occurring in Zimbabwe, about 101 are used for medicinal purposes. Many communities in the country, mainly in peri-urban, rural and marginalized areas with limited access to healthcare facilities, rely on traditional medicines as their primary healthcare. The study reviewed research studies undertaken on Zimbabwe’s Fabaceae species during 1959 to 2022. Information was gathered from literature sourced from Google Scholar, Science Direct, Scopus, PubMed, books, dissertations, theses and scientific reports. This study showed that 101 species are traditionally used to manage human and animal diseases in Zimbabwe. The genera with the highest number of medicinal uses are *Indigofera*, *Senna*, *Albizia*, *Rhynchosia* and *Vachellia*. Species of these genera are used as traditional medicines against 134 medical conditions, mainly gastrointestinal conditions, female reproductive conditions, respiratory conditions and sexually transmitted infections. Shrubs (39.0%), trees (37.0%) and herbs (18.0%) are the primary sources of traditional medicines, while roots (80.2%), leaves (36.6%), bark (27.7%) and fruits (8.9%) are the most widely used plant parts. Many of Zimbabwe’s Fabaceae species used as sources of traditional medicines have been assessed for their phytochemical and pharmacological properties, corroborating their medicinal uses. However, there is a need to unravel the therapeutic potential of the family through further ethnopharmacological research focusing on toxicological studies, in vitro and in vivo models, biochemical assays and pharmacokinetic studies.

## 1. Introduction

The Fabaceae (Leguminosae), often referred to as the bean, legume or pea family, is the third largest plant family after the Asteraceae and Orchidaceae in terms of plant species numbers [1]. The Fabaceae family consists of approximately 770 genera and 19,500 species [1,2] recorded in almost all of the biomes in the world except Antarctica and the high Arctic [3]. Research has shown that the success of the family in dominating in several hospitable and disturbed habitats is ascribed to the ability of the species to fix atmospheric nitrogen, thus allowing the plant species to grow in nutrient-poor soils [4,5,6]. Recent morphological and molecular research has supported that the Fabaceae family is a monophyletic family [2,7]. However, the Fabaceae family is divided into six subfamilies, namely the Caesalpinioideae (148 genera and 4400 species), Cercidoideae (12 genera and 335 species), Detarioideae (84 genera and 760 species), Dialiodeae (17 genera and 85 species), Duparquetioideae (monotypic genus) and Faboideae (or Papilionoideae) (503 genera and 14,000 species) [2]. Members of the Fabaceae family include trees, shrubs, subshrubs, woody lianas, climbing annuals, herbs and aquatics [8]. The flowers are asymmetric, bilaterally symmetric or radially symmetric, and are pollinated by bats, birds and insects [9]. The leaves of the majority of species belonging to the Fabaceae family are compound, double-compound or trifoliolate, sometimes with a swollen leaf base, a superior ovary with one locular, and the fruit is usually a two-valved, dehiscent pod that is rarely fleshy but is sometimes indehiscent and occasionally breaking into segments [10,11]. 

The majority of the members of the Fabaceae family are culturally and economically important throughout the world, and are used as sources of traditional medicines, food, timber, garden ornamentals, dyes, fibres, fuels, gums and insecticides [6,12,13]. The role played by Fabaceae species in the provision of ecosystem services and goods that support human wellbeing and survival have been highlighted in some studies conducted in different countries of the world [14,15]. Many members of this family have been widely studied for their bioactive chemical constituents such as phenolic acids, flavonoids, lectins, saponins, alkaloids and carotenoids [16]. Pharmacological studies have shown that some species exhibit potent anticancer, antioxidant, antimicrobial, anti-inflammatory, analgesic, antiulcer, antidiabetic, antirheumatic, cytotoxic and antiparasitic activities, among others [16,17,18]. Therefore, extensive phytochemical and pharmacological evaluations of some of the utilized Fabaceae species may lead to the discovery and development of novel pharmaceutical products, functional food ingredients and cosmetic products. Despite the discovery of several secondary metabolites in the Fabaceae, this family has attracted disproportionately little attention in the context of ethnopharmacological research. It is, therefore, within this context that this study was undertaken, with the aim of exploring and documenting the ethnomedicinal knowledge of Zimbabwe. Such a synthesis identified the gaps in knowledge on the therapeutic potential of the Fabaceae species and may also provide helpful information on ethnopharmacological research areas that require further research.

## 2. Materials and Methods

**Figure 1:**
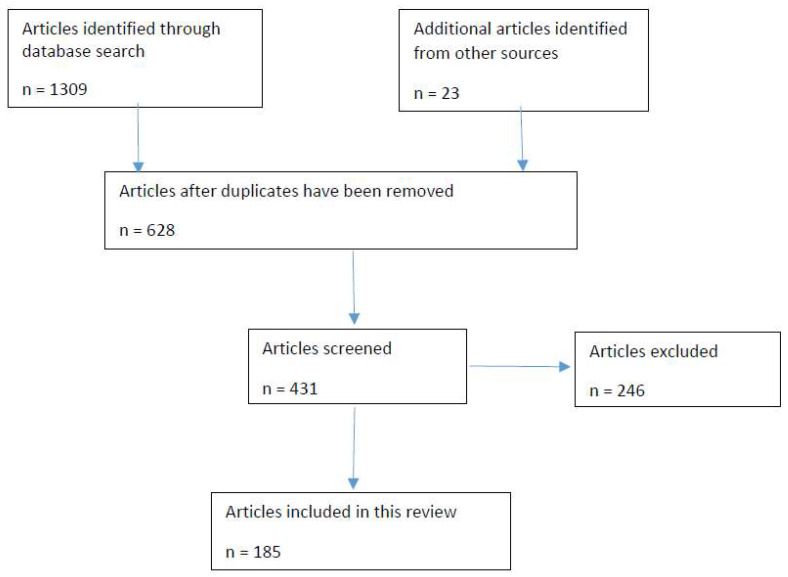
Flow diagram showing the identification and screening of the articles used in this review.

A literature search on Fabaceae species used as traditional medicines in Zimbabwe was conducted from September 2021 to November 2022. This information was retrieved from different online databases such as BioMed Central, Web of Science, Springerlink, Google Scholar, Scielo, PubMed, Science Direct, ACS Publications, Scopus and JSTOR. In addition, theses, dissertations, book chapters, books and scientific reports were retrieved from the libraries of the University of Fort Hare (UFH) in South Africa and the National Herbarium (SRGH) in Harare, Zimbabwe. Keywords and terminologies such as Zimbabwe, ethnobotany, ethnomedicine, ethnopharmacology, indigenous, medicine, phytomedicine, traditional medicine, Zimbabwean Fabaceae, Zimbabwean Leguminosae, medicinal Fabaceae, medicinal Leguminosae, Zimbabwean traditional medicine, Fabaceae and Leguminosae were used to search for relevant articles as shown in the PRISMA flow diagram (Figure 1). From each article, the following information was collected: the scientific names of the plant species, their growth form, plant part(s) used, methods of preparation and medicinal uses. The medicinal use categories were classified according to the Economic Botany Data Collection Standard [19]. The scientific names of the Fabaceae species from the original data sources were updated to the recently accepted names according to the Plants of the World Online website [20]. The Fabaceae subfamilies were updated following the classifications of the “Legume Phylogeny Working Group”, which presently recognizes six subfamilies: Caesalpinioideae, Cercidoideae, Detarioideae, Dialioideae, Duparquetioideae and Papilionoideae [2]. 

## 3. Results and Discussion

### 3.1. Medicinal Plant Diversity

This study recorded 101 species traditionally used to manage and treat human and animal diseases in Zimbabwe (Table 1). Of these, 91 species are indigenous to Zimbabwe (90.1%), while nine species are exotic (8.9%), either naturalized as weeds or cultivated in home gardens and agricultural fields as ornamentals, fodder or food plants. The subfamilies Caesalpinoideae and Faboideae are dominant, with 54 species (53.5%) and 55 species (54.5%), respectively, and the remaining two species belonging to the Cercidoideae. Therefore, 101 species (15.2%) out of 665 species of the Fabaceae family known to occur in Zimbabwe [21] are used as sources of traditional medicines. A similar study by Van Wyk [6] showed that 338 species out of 1748 Fabaceae species (19.3%) are used as traditional medicines in southern Africa. Similar findings have been reported in Thailand, where 261 species out of 688 Fabaceae species are used as sources of traditional medicines [22]. Macêdo et al. [23] and Sutjaritjai et al. [24] argued that the prominence of Fabaceae taxa in traditional pharmacopoeia throughout the world is possibly associated with the wide distribution of the family, as the different growth forms of the species grow in many types of habitats and vegetation, and therefore are available in all seasons.

**Figure 2:**
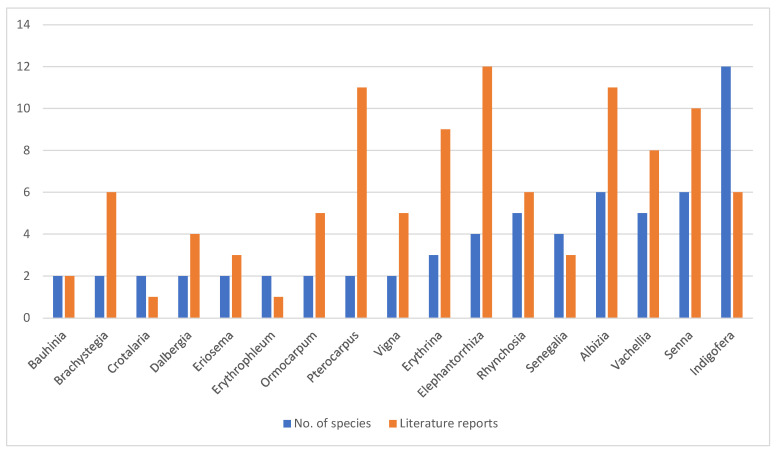
Numbers of species and reports of the use of Fabaceae genera with medicinal uses in Zimbabwe.

*Indigofera* is the genus with the highest number of medicinal Fabaceae species (12 species), followed by *Senna* (six species), and *Albizia*, *Rhynchosia* and *Vachellia* with five species each (Figure 2). However, the genera associated with the highest number of records in the literature are *Elephantorrhiza* (12 records), *Pterocarpus* (11 records), *Senna* (10 records), *Albizia* and *Erythrina* (nine records each) and *Vachellia* with eight records (Figure 2). The number of medicinal species found in each genus is significantly correlated to the total number of species in each genus in Zimbabwe (*p* < 0.01, r = 0.772). These results are consistent with those observed by Anorld et al. [49], who recorded 11 medicinal species of *Albizia*, followed by *Rhynchosia* (12 species), *Senna* (17 species), and *Indigofera* and *Vachellia* with 32 species each. In Botswana, Hedberg and Staugård [50] argued that one *Senna* species, followed by *Albizia* (three species), *Rhynchosia* (four species), *Indigofera* (five species) and *Vachellia* (six species) were used as traditional medicines in that country. Moreover, several species of *Albizia*, *Elephantorrhiza*, *Erythrina*, *Senna* and *Vachellia* are included in the monograph *Medicinal Plants of South Africa*, with detailed information on their botany, medicinal uses, preparation, dosage, active ingredients and pharmacological effects [51].

### 3.2. Growth Habit and Parts Used

**Figure 3:**
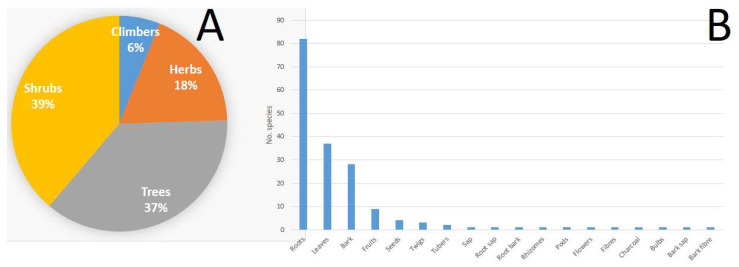
Characteristics of Fabaceae species used as traditional medicines in Zimbabwe. (**A**): Growth habit as a pie diagram and (**B**): Plant parts used presented as a bar chart.

Shrubs (39.0%), followed by trees (37.0%) and herbs (18.0%), are the primary sources of the medicinal Fabaceae species in Zimbabwe (Figure 3A). The plant parts used for traditional medicine preparations include bark, bark fibre, bark sap, bulbs, charcoal, fibre, flowers, fruits, leaves, pods, rhizomes, roots, root bark, root sap, sap, seeds, tubers and twigs (Table 1). The roots are the most frequently used (81 species), followed by leaves (37 species), bark (28 species), fruits (nine species), seeds (four species), twigs (three species) and tubers (two species), with the rest of the plant parts represented by a single species each (Figure 3B). However, harvesting the roots of herbaceous plants for medicinal purposes is not sustainable, as it threatens the survival of these plants used to treat human and animal diseases. It is well recognized by conservationists that medicinal plants primarily valued for their roots and those which are intensively harvested for their bark often tend to be the most threatened by overexploitation [52,53]. *Afzelia quanzensis*, *Baikiaea plurijuga*, *Dalbergia melanoxylon* and *Pterocarpus angolensis* are listed in the Zimbabwean Red Data List, as these four species are threatened with extinction mainly due to overexploitation as sources of timber for construction or wood carving [54]. 

### 3.3. Usage Categories with High Numbers of Reports

The 134 medical reports of Fabaceae species in Zimbabwe (Table 1 and Table 2) are classified into 19 major health disorder categories following the International Classification of Primary Care’s classification system [19]. Most use records are in the categories of gastrointestinal problems (92 usage reports) and female reproductive problems (58 usage reports) (Table 2). Similarly, gastrointestinal problems, reproductive problems in women, respiratory problems and sexually transmitted infections (Table 2) are treated with the highest number of species. The categories of gastrointestinal problems, reproductive problems, respiratory problems and sexually transmitted infections are among the 10 major causes of death in Zimbabwe [55]. Muchandiona [56] argued that the prevalence of gastrointestinal disorders and respiratory infections is due to poor solid waste management by the local councils in Zimbabwe, which has worsened over the years. Similarly, gastrointestinal disorders, such as diarrhoea and dysentery, are also a major concern in neighbouring countries such as Mozambique [57,58,59] and South Africa [60,61,62]. Therefore, gastrointestinal problems are among the most common reasons local people use traditional medicines and consult traditional healers [57,59,60,61,62]. 

**Figure 4:**
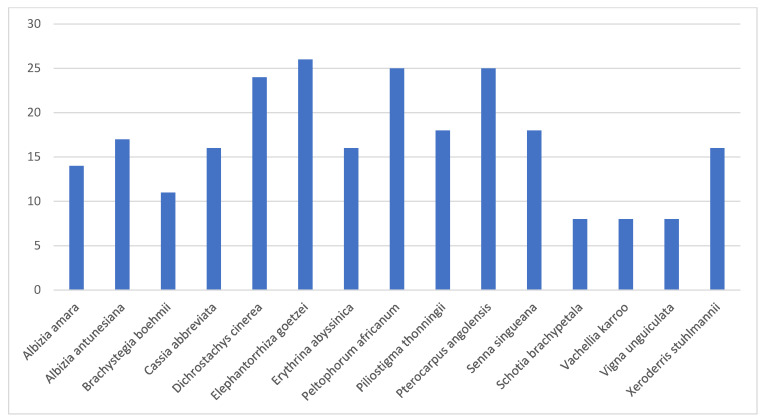
Fabaceae species with eight or more usage reports cited in at least four references.

Fifteen medicinal species are known to have more than eight usage reports (Figure 4). These species included Albizia amara, Albizia antunesiana, Brachystegia boehmii, Cassia abbreviate, Dichrostachys cinerea, Elephantorrhiza goetzei, Erythrina abyssinica, Peltophorum africanum, Piliostigma thonningii, Pterocarpus angolensis, Schotia brachypetala, Senna singueana, Vachellia karroo, Vigna unguiculata and Xeroderris stuhlmannii. Some of these plant species are widely used as sources of traditional medicines in Angola [63], Botswana [50,64], Eswatini [65], Malawi [66,67], Mozambique [58,68], Namibia [69,70], South Africa [71,72] and Zambia [73,74]. The importance of these species as sources of traditional medicines is documented in the monographs Medicinal and Magical Plants of Southern Africa: An Annotated Checklist [49], Plant Resources of Tropical Africa 11: Medicinal Plants 1 and 2 [75,76] and Medicinal Plants of South Africa [51]. Research by Van Wyk [77] revealed that Colophospermum mopane, Dichrostachys cinerea and Vachellia karroo are commercially exploited in local, regional or international trade in eastern, southern and western Africa.

### 3.4. Phytochemistry and Pharmacological Properties of Fabaceae Species

The Fabaceae species used as sources of traditional medicines in Zimbabwe are rich in chemical constituents (Table 3). The majority of these species are characterized by flavonoids (57.4%), followed by terpenoids (42.6%), tannins (40.6%), saponins (34.7%), phenolics (30.7%) and alkaloids (28.7%) (Table 3). Research by Wink [78] showed that the main secondary metabolites of the Fabaceae family include alkaloids, non-protein amino acids, cyanogens, peptides, phenolics, polyketides and terpenoids. This author argued that these secondary metabolites serve as defence compounds against herbivores and microbes and also serve as signal compounds to attract pollinating and fruit-dispersing animals. Fabaceae species used as traditional medicines and food plants are characterized by nutrients such as proteins, lipids, carbohydrates, mineral elements, fatty acids, amino acids, fibres and vitamins, which are important for animal and human health [79,80]. The majority of documented species have several proven pharmacological activities (Table 3) such as inhibition of the acetylcholinesterase enzyme, and anticancer, antidiabetic, antifertility, anthelmintic, antiamoebic, anti-inflammatory, antimicrobial, antioxidant, antiparasitic, cytotoxic, hepatoprotective, hypoglycaemic and immunomodulatory effects. Despite the discovery of several secondary metabolites in the Fabaceae family, its species have attracted disproportionately little attention in the context of ethnopharmacological research over the years. The relative importance of the Fabaceae species as medicinal plants is demonstrated by the fact that about 10% of the species documented in this study are commercially important. The species that are commercially developed with potential to be developed into health products or pharmaceutical drugs and are regularly traded on the international markets include Abrus precatorius, Albizia adianthifolia, Cajanus cajan, Colophospermum mopane, Dichrostachys cinerea, Lessertia frutescens, Senna italica, Senna occidentalis, Tamarindus indica, Vachellia karroo and Vachellia nilotica [77,81].

## 4. Conclusions

This review is a compilation of literature sources on the Fabaceae species used as traditional medicines in Zimbabwe, providing an important repository of ethnopharmacological data required for future studies. The Fabaceae family is characterized by several species used as traditional medicines for the treatment and management of different ailments and diseases. The literature search showed that there is a paucity of information on the cultural practices associated with usage of Fabaceae species, including information on their dosages and administration. Therefore, there is a need for ethnobotanical research into and documentation of the cultural value of the Fabaceae species in Zimbabwe. Fabaceae species that are exotic to Zimbabwe are also used as sources of traditional medicines, corroborating the general observation that traditional pharmacopoeias are not static social institutions but fluid and dynamic, characterized by the addition of exotic plant species as herbal medicines. 

Several Fabaceae species used as traditional medicines are known to contain bioactive compounds which have demonstrated diverse pharmacological properties against several disease-causing pathogens. Plant extracts and phytochemical compounds isolated from Fabaceae species have shown inhibition of the acetylcholinesterase enzyme and many other properties, such as antitumor, antidiabetic, antifertility, anthelmintic, antiamoebic, anti-inflammatory, antimicrobial, antioxidant, antiparasitic, cytotoxic, hepatoprotective, hypoglycaemic and immunomodulatory. However, the majority of the studied biological activities have mainly been in vitro assays, while clinical and in vivo studies are lacking. It is recommended that the unstudied biological activities of the medicinal species should be investigated to unravel the therapeutic potential of the considered Fabaceae species, using both in vitro and in vivo models. Furthermore, the toxicological properties of these species should be evaluated and the mechanism of action of the identified phytochemicals should be elucidated based on their pharmacological properties. 

## Figures and Tables

**Table 1:** Medicinal Fabaceae plants of Zimbabwe.

Plant Taxa	Habit	Parts Used	Medicinal Uses	Literature Records	References
*Abrus precatorius* L. subsp. *africanus* Verdc. ^+1^	Climber	Leaves, roots and seeds	Bilharzia (schistosomiasis), sexually transmitted infections (STIs) and lucky charms	4	[25,26,27,28]
*Aeschynomene mimosifolia* Vatke ^1^	Shrub	Roots	Chest pains and headache	1	[25]
*Afzelia quanzensis* Welw. ^2^	Tree	Bark, fruits and root bark	Bloated stomach, blood pressure, depressed fontanelle, haemorrhoids and stomach problems, lucky charms and painful udders in cattle	2	[25,28]
*Albizia amara* (Roxb.) Boivin ^2^	Tree	Bark, leaves and roots	Aphrodisiac, constipation, diarrhoea, dilating the birth canal, dysentery, oedema, painful placenta, palpitations, pneumonia, purgative, stomach problems, tuberculosis (TB), warts, and protection against witchcraft	5	[25,28,29,30,31]
*Albizia adianthifolia* (Schumach.) W.Wight ^2^	Tree	Roots	Ethnoveterinary medicine	2	[32,33]
*Albizia anthelmintica* (A.Rich.) Brongn. ^2^	Shrub	Bark	Wounds	1	[25]
*Albizia antunesiana* Harms ^2^	Tree	Bark, bark sap, leaves and roots	Abdominal pains, aphrodisiac, bilharzia, constipation, depressed fontanelle, diarrhoea, gonorrhoea, infertility in women, menstrual problems, painful legs, painful uterus, preventing abortion, purgative, sexually transmitted diseases (STDs), sore eyes, sore throat and swollen legs	8	[25,26,27,28,34,35,36,37]
*Albizia tanganyicensis* Baker ^2^	Tree	Bark and roots	Cough and swollen legs	1	[25]
*Albizia versicolor* Welw. ex Oliv. ^2^	Tree	Roots	Erectile dysfunction, infertility in men and sexual impotence	3	[25,28,29]
*Arachis hypogaea L. ^1^	Herb	Leaves	Cataracts, infertility in women and sore eyes	1	[25]
*Baikiaea plurijuga* Harms ^2^	Tree	Bark	Bloated stomach and haemorrhoids	1	[28]
*Bauhinia galpinii* N.E.Br. ^3^	Shrub	Roots and seeds	Infertility and menstrual problems	2	[25,28]
*Bauhinia petersiana* Bolle ^3^	Tree	Roots	Depressed fontanelle, infertility in women, menstrual problems and preventing witchcraft	2	[25,28]
*Bobgunnia madagascariensis* (Desv.) J.H. Kirkbr. & Wiersema ^1^	Tree	Fruits, pods and roots	Abdominal pains, convulsions, diarrhoea, emetic, earache, headache, infertility in men and women, oedema, stomach problems, syphilis and wounds	3	[25,28,34]
*Bolusanthus speciosus* (Bolus) Harms ^1^	Tree	Leaves	Bile emesis and emetic	1	[25]
*Brachystegia boehmii* Taub. ^2^	Tree	Bark, leaves, roots and twigs	Abdominal pains, antivenom, back pain, cataracts, heart problems, mental problems, sore eyes, STIs, toothache, constipation and lumbago in ruminants	5	[25,28,34,36,37]
*Brachystegia spiciformis* Benth. ^2^	Tree	Bark, fibre and roots	Constipation, diarrhoea, mental problems, pain, sore eyes and wounds	3	[25,28,38]
*Burkea africana* Hook. ^2^	Tree	Bark, leaves and roots	Abdominal pains, anti-inflammatory, bilharzia, cancer, diarrhoea, fever, immune system booster, infections, oedema and ulcers	3	[25,26,39]
* *Cajanus cajan* (L.) Huth. ^1^	Shrub	Leaves	Earache	1	[25]
*Cassia abbreviata* Oliv. ^2^	Tree	Bark, fruits, roots and twigs	Abdominal pains, abortifacient, aphrodisiac, backache, bilharzia, cancer, constipation, diarrhoea, gonorrhoea, hydrocele, lucky charms, malaria, menstrual problems, stomach pains, STDs and venereal diseases	11	[25,26,27,28,34,35,36,37,38,40,41,42]
*Colophospermum mopane* (J.Kirk ex Benth.) J.Léonard ^2^	Tree	Bark, charcoal, leaves	Constipation, diarrhoea, snake bite and diarrhoea in cattle	3	[25,28,38]
*Crotalaria laburnifolia* L. ^1^	Herb	Roots	Cough	1	[25]
*Crotalaria rogersii* Bak.f. ^1^	Herb	Roots	Infertility in women and lucky charms	1	[25]
*Dalbergia melanoxylon* Guill. & Perr. ^1^	Shrub	Bark	Asthma and wounds	3	[37,38,43]
*Dalbergia nitidula* Welw. ex Bak. ^1^	Shrub	Bark and roots	Aphrodisiac, driving away bad spirits, preventing witchcraft and ulcers	1	[25]
*Dalbergiella nyasae* Bak.f. ^1^	Tree	Leaves and roots	Tetanic contractions and driving away maggots from wounds	1	[25]
*Dichrostachys cinerea* (L.) Wight & Arn. ^2^	Tree	Leaves, fruits or roots	Abdominal pains, antivenom, backache, cancer, colic, contraceptive, cough, depressed fontanelle, diarrhoea, dilating the birth canal, epistaxis, infertility in women, influenza, inducing labour, mental problems, oedema, postpartum, scabies, scorpion stings, STDs, stomach problems, syphilis, urticaria (skin swellings) and wounds	8	[25,28,29,31,34,35,38,40]
*Dolichos kilimandscharicus* Taub. ^1^	Herb	Tubers	Abdominal pains, antiemetic, constipation, diarrhoea and measles	1	[25]
*Elephantorrhiza burkei* Benth. ^2^	Shrub	Roots	Antiemetic, constipation, increasing blood in the body and postpartum conditions	1	[25]
*Elephantorrhiza elephantina* (Burch.) Skeels ^2^	Shrub	Roots	Abdominal pains, aphrodisiac, infertility in women, postpartum conditions and reducing the size of the vagina	3	[25,34,44]
*Elephantorrhiza goetzei* (Harms) Harms ^2^	Shrub	Bark, rhizomes or roots	Abdominal pains, anthelmintic, backache, bilharzia, bloating, blood pressure, boosting appetite, constipation, cough, depressed fontanelle, diarrhoea, dilating the birth canal, erectile function, fever, gonorrhoea, heart pains, human immunodeficiency virus/acquired immunodeficiency syndrome (HIV/AIDS) opportunistic infections, increasing blood in the body, infertility, influenza, malaria, painful uterus, postpartum conditions, rash, STIs and stomach problems	11	[25,26,27,28,31,34,35,36,37,38,41]
*Elephantorrhiza suffruticosa* Schinz ^2^	Shrub	Roots	Constipation and diarrhoea	1	[25]
*Eriosema englerianum* Harms ^1^	Shrub	Leaves and roots	Aphrodisiac, backache, bilharzia, blood pressure, infertility in women, menstrual problems, painful uterus, venereal disease and wasting in infants	3	[25,27,31]
*Eriosema rhynchosioides* Bak. ^1^	Shrub	Roots	Tonic	1	[25]
*Erythrina abyssinica* Lam. ex DC. ^1^	Tree	Bark, leaves and roots	Abdominal pains, backache, bilharzia, blood pressure, cough, cracked heels, diarrhoea, gonorrhoea, lucky charms, mental problems, STDs, stop bad dreams, wasting in infants, wounds and wounds in the mouth	9	[25,26,27,28,29,31,36,37,40]
*Erythrina livingstoniana* Bak. ^1^	Tree	Roots	Haematuria	1	[25]
*Erythrina* spp. ^1^		Roots	Backache and manic disorders	1	[29]
*Erythrophleum africanum* (Benth.) Harms ^2^	Tree	Bark	Stomach pains	1	[25]
*Erythrophleum suaveolens* (Guill. & Perr.) Brenan ^2^	Tree	Bark	Preventing witchcraft	1	[25]
*Flemingia grahamiana* Wight & Arry ^1^	Herb	Roots	Diarrhoea	1	[25]
*Grona barbata* (L.) H.Ohashi & K.Ohashi ^1^	Herb	Roots	Abortifacient, dilating the birth canal, epilepsy, preventing abortion, postpartum conditions, sore eyes and wasting in infants	1	[25]
*Indigofera antunesiana* Harms ^1^	Shrub	Roots	Menstrual problems	1	[25]
*Indigofera arrecta* Hochst. ex A.Rich. ^1^	Shrub	Leaves and roots	Abdominal pains, abortifacient, convulsions, diuretic, gonorrhoea, infertility, purgative, sore eyes, stomach pains and de-ticking dogs	2	[25,35]
*Indigofera astragalina* DC. ^1^	Herb	Roots	Dizziness	1	[25]
*Indigofera demissa* Taub. ^1^	Herb	Roots	Abortifacient	1	[25]
*Indigofera hilaris* Eckl. & Zeyh. ^1^	Herb	Roots	Painful legs	1	[25]
*Indigofera hirsuta* L. ^1^	Shrub	Roots	Dizziness	1	[34]
*Indigofera rhynchocarpa* Bak. ^1^	Shrub	Roots	Abdominal pains and menstrual problems	1	[25,34]
*Indigofera setiflora* Baker ^1^	Herb	Roots	Diarrhoea and stomach problems	3	[28,37,43]
*Indigofera spicata* Forssk. ^1^	Herb	Roots	Panacea	1	[25]
*Indigofera vicioides* Jaub. & Spach. ssp. *rogersii* (R.E.Fr.) Schrire ^1^	Shrub	Roots	Depressed fontanelle	1	[25]
*Indigofera wildiana* J.B.Gillett ^1^	Shrub	Roots	Preventing abortion	1	[25]
*Indigofera* spp. ^1^	Shrub	Leaves and roots	Abdominal pains, antenatal conditions, chest pains, coughs, driving away bad spirits and infertility in women	2	[25,34]
*Julbernardia globiflora* (Benth.) Troupin ^2^	Tree	Bark, bark fibre, leaves and roots	Constipation, diarrhoea, reducing the size of the vagina, snakebite, sore eyes, stomach problems and diarrhoea in cattle	3	[25,28,38]
* *Lessertia frutescens* (L.) Goldblatt & J.C.Manning ^1^ (Syn. *Sutherlandia frutescens* (L.) W.T,Aiton)	Shrub	Roots	Analgesia, cancer, colds, diabetes, fever, influenza and haemorrhoids	1	[39]
* *Leucaena leucocephala* (Lam.) DeWit ^2^	Shrub	Bark, leaves and seeds	Colds, influenza and TB	1	[45]
*Macrotyloma densiflorum* (Welw. ex Bak.) Verdc. ^1^	Shrub	Leaves	Abdominal pains	1	[25]
*Mucuna coriacea* Baker ^1^	Climber	Roots	Bilharzia	1	[26]
*Mundulea sericea* (Willd.) A.Chev. ^1^	Shrub	Roots	Infertility and sexual impotence	2	[25,29]
*Neorautanenia mitis* (A.Rich.) Verdc. ^1^	Climber	Bulbs	Fever and de-ticking dogs	2	[25,28]
*Ormocarpum kirkii* S.Moore ^1^	Tree	Leaves	Depressed fontanelle, dilating the birth canal and stomach pains	3	[28,31,34]
*Ormocarpum trichocarpum* (Taub.) Engl. ^1^	Shrub	Leaves and roots	Allergies, depressed fontanelle, prolonged labour and stomach problems	2	[25,29]
*Peltophorum africanum* Sond. ^2^	Tree	Bark, leaves and roots	Abdominal pains, bilharzia, blood purification, chest pains, diaphoretic, diarrhoea, diuretic, driving away evil spirits, dropsy, eye problems, headache, infertility in women, laxative, mental problems, nausea, oedema, panacea, preventing abortion, sore eyes, sore throat, STDs, STIs, syphilis, toothache and venereal diseases	10	[25,26,27,29,34,35,36,37,38,46]
*Pericopsis angolensis* (Baker) Meeuwen ^1^	Tree	Bark or roots	Abdominal pains, antiemetic, backache, cancer, cough, diarrhoea, dyspnoea, oedema, sore throats and wounds	3	[25,28,38]
* *Phaseolus vulgaris* L. ^2^	Shrub	Roots	Bilharzia and postpartum conditions	2	[26,34]
*Philenoptera violacea* (Klotzch) Schrire ^1^	Tree	Roots	Diarrhoea	1	[38]
*Piliostigma thonningii* (Schumach.) Milne-Redh. ^2^ (Syn. *Bauhinia thonningii* Schumach.)	Tree	Bark, fruits, leaves and roots	Abdominal pains, antivenom, bilharzia, constipation, convulsions, cough, diarrhoea, dropsy, emetic, immune booster, influenza, menstrual problems, painful legs, painful uterus, postpartum conditions, stomach problems and ketosis in cattle	7	[25,26,28,29,36,37,38]
*Pseudarthria hookeri* Wight & Arn. ^1^	Herb	Leaves and roots	Bilharzia and diarrhoea	1	[25]
*Pterocarpus angolensis* DC. ^1^	Tree	Bark, flowers, fruits, leaves, roots and sap	Abdominal pains, anaemia, aphrodisiac, asthma, backache, bilharzia, body pains, cataract, cough, depressed fontanelle, diarrhoea, earache, haematuria, infertility in women, kwashiorkor, lameness, menstrual problems, pelvic inflammation, ringworm, sore eyes, stomach problems, TB, ulcers and venereal diseases and sore eyes in animals	11	[25,26,27,28,29,31,35,36,37,38,46]
*Pterocarpus rotundifolius* (Sond.) Druce ^1^	Tree	Root sap	Sore eyes	1	[25]
*Pterolobium stellatum* (Forssk.) Brenan ^2^	Climber	Roots	Augmenting labour and depressed fontanelle	2	[31,38]
*Rhynchosia insignis* (O.Hoffm.) R.E.Fr. ^1^	Herb	Roots	Abdominal pains, depressed fontanelle and dropsy	1	[25]
*Rhynchosia minima* (L.) DC. ^1^	Herb	Roots	Boils and skin infections	1	[47]
*Rhynchosia monophylla* Schltr. ^1^	Herb	Roots	Postpartum conditions	1	[25]
*Rhynchosia resinosa* (Hochst. ex A.Rich.) Bak. ^1^	Climber	Leaves and roots and twigs	Abdominal pains, diabetes mellitus, dilating the birth canal, expel maggots from wounds, high blood pressure, infertility and menstrual problems	3	[25,31,34]
*Rhynchosia* spp. ^1^	Herb	Roots	Diarrhoea	1	[38]
*Schotia brachypetala* Sond. ^2^	Tree	Bark, leaves and roots	Depressed fontanelle, diarrhoea, dysentery, epistaxis, oedema, stomach problems, swellings and ulcers	4	[25,28,34,38]
*Senegalia ataxacantha* (DC.) Kyal. & Boatwr. ^2^ (Syn. *Acacia ataxacantha* DC.)	Shrub	Roots	Abdominal pains, constipation and preventing witchcraft	1	[25]
*Senegalia chariessa* (Milne-Redh.) Kyal. & Boatwr. ^2^ (Syn. *Acacia chariessa* Milne-Redh.)	Shrub	Roots	Antenatal, blood purification and postpartum	2	[25,29]
*Senegalia mellifera* (Benth.) Seigler & Ebinger ^2^ (Syn. *Acacia mellifera* Benth.)	Shrub	Bark	Aphrodisiac	2	[25,28]
*Senegalia nigrescens* (Oliv.) P.J.H.Hurter ^2^ (Syn. *Acacia nigrescens* Oliv.)	Tree	Roots	Convulsions	1	[25]
*Senna didymobotrya* (Fresen.) H.S.Irwin & Barneby ^2^ (Syn. *Cassia didymobotrya* Fresen.)	Shrub	Roots	Convulsions and mental problems	1	[25]
*Senna italica* Mill. ^2^ (Syn. *Cassia italica* (Mill.) F.W.Andr.)	Shrub	Roots	Abdominal pains, bilharzia, bronchitis, colic, haemorrhoids and sore eyes	3	[25,26,29]
* *Senna occidentalis* (L.) Link ^2^ (Syn. *Cassia occidentalis* L.)	Herb	Roots	Sore throats and tonsillitis	1	[28]
*Senna petersiana* (Bolle) Lock ^2^ (Syn. *Cassia petersiana* Bolle)	Tree	Roots	Bilharzia	1	[26]
*Senna singueana* (Delile) Lock ^2^ (Syn. *Cassia singueana* Delile)	Shrub	Bark, leaves and roots	Abdominal pains, antiemetic, bilharzia, constipation, dropsy, herpes, infertility in women, malaria, menstrual problems, painful uterus, postpartum, preventing still birth, preventing bad luck, sores, sore eyes, STDs, syphilis and venereal diseases	7	[25,26,27,28,34,38,40]
* *Senna septemtrionalis* (Viv.) H.S.Irwin & Barneby ^2^ (Syn. *Cassia septemtrionalis* Viv.)	Shrub	Roots	Malaria	1	[41]
*Sesbania* spp. ^1^	Shrub	Seeds	Fever	1	[34]
*Sphenostylis erecta* (Baker f.) Hutch. ex Baker f. ^1^ (Syn. *Sphenostylis marginata* E.Mey. ssp. *erecta* (Baker f.) Verdc.)	Shrub	Roots	Abdominal pains, bile emesis, constipation, diarrhoea, fever, oedema and wasting away in infants	2	[25,38]
* *Tamarindus indica* L. ^2^	Tree	Fruits and roots	Sore throat and venereal diseases	1	[25]
*Tephrosia radicans* Welw. ^1^	Shrub	Roots	Sore eyes and toothache	1	[25]
*Tylosema fassoglense* (Kotschy ex Schweinf.) Torre & Hillc. ^2^	Climber	Bark, roots and tubers	Abdominal pains, diarrhoea, pneumonia, retained placenta, stomach problems and venereal diseases	2	[25,28]
*Vachellia amythethophylla* (Steud. ex A.Rich.) Kyal. & Boatwr. ^2^ (Syn. *Acacia amythethophylla* Steud. ex A.Rich.)	Shrub	Roots	Antidote for snakebites, convulsions, driving away evil spirits, excessive sweating, infertility in women, mental problems and painful uterus	1	[25]
*Vachellia karroo* (Hayne) Banfi & Galasso ^2^ (Syn. *Acacia karroo* Hayne)	Tree	Fruits and roots	Aphrodisiac, bilharzia, body pains, convulsions, dizziness, gonorrhoea, syphilis and killing parasites in fowl runs	6	[25,26,27,36,37,43]
*Vachellia nilotica* (L.) P.J.H.Hurter & Mabb. ^2^ (Syn. *Acacia nilotica* L.)	Shrub	Fruits and roots	STDs	1	[40]
*Vachellia rehmanniana* (Schinz) Kyal. & Boatwr. ^2^ (Syn. *Acacia rehmanniana* Schinz)	Tree	Bark and roots	Bloated stomach, headaches and pneumonia	1	[25,28]
*Vachellia sieberiana* (DC.) Kyal. & Boatwr. ^2^ (Syn. *Acacia sieberiana* DC.)	Shrub	Roots	Antiseptic	1	[25]
*Vigna nuda* N.E.Br. ^1^	Herb	Roots	Chest pains and cough	1	[34]
# *Vigna unguiculata* (L.) Walp.^1^	Shrub	Roots and seeds	Anaemia, antivenom, bilharzia, chest pains, constipation, epilepsy, menstrual problems and antidote for snakebites	4	[25,26,27,28]
*Xeroderris stuhlmannii* (Taub.) Mendonça & E.P.Sousa ^1^	Tree	Bark, leaves or roots	Abdominal pains, anaemia, antiabortifacient, back pains, cancer, diarrhoea, headache, infertility in men, malaria, menstrual problems, pneumonia, stomach problems, toothache, venereal diseases, wounds and ethnoveterinary medicine	4	[25,28,38,48]
*Zornia glochidiata* Rchb. ex DC. ^1^	Herb	Roots	Dilating the birth canal, preventing abortion and venereal diseases	1	[25]

* *=* Exotic; # *=* cultivated or collected from semi-natural landscapes; ^+^ = Fabaceae subfamilies: ^1^ = Faboideae, ^2^ = Caesalpinoideae, ^3^ = Cercidoideae.

**Table 2:** Major disease categorises and Fabaceae species used as traditional medicines in Zimbabwe.

Disease Category	Species	Usage Records
Antenatal and postpartum conditions	15	23
Antivenom	8	11
Back pain	10	14
Bilharzia	19	27
Charms and ritual objects	15	26
Convulsions and epilepsy	9	18
Depressed fontanelle	12	24
Ethnoveterinary medicine	10	20
Fever and malaria	11	19
Gastrointestinal problems	45	92
Mental problems	10	16
Oedema	11	19
Reproductive problems in men	18	36
Reproductive problems in women	27	58
Respiratory problems	26	39
Sexually transmitted infections	20	31
Skin problems	8	16
Sore eyes	15	27
Sores and wounds	11	24

**Table 3:** Phytochemistry and pharmacological properties of Fabaceae species used as traditional medicines in Zimbabwe.

Species	Phytochemistry	Pharmacological Activities	References
*Abrus precatorius*	Alkaloids, esters, flavonoids, organic acids, phenolics, steroids and terpenoids	Antidiabetic, antifertility, anti-inflammatory, antimicrobial, antioxidant, antiparasitic, antiprotozoal, antitumor, immunomodulatory and insecticidal	[82]
*Aeschynomene mimosifolia*	Flavonoids	Cytotoxicity	[83]
*Afzelia quanzensis*	Fatty acids	Antifungal	[84]
*Albizia amara*	Alkaloids, glycosides, flavonoids, phenols, quinones, saponins, sterols, tannins and terpenoids	Analgesic, antiarthritic, antibacterial, antifungal, antiviral, anticancer, antihyperlipidemic, anti-inflammatory, antioxidant and hepatoprotective	[85,86,87]
Albizia adianthifolia	Apocarotenoids, chalcone, dipeptide, elliptocytes, fatty acids, flavonoids, histamine, imidazolyl carboxylic acid, prosapogenins, saponins, steroids, triterpenoids and volatile oils	Acetylcholinesterase enzyme inhibitory, anthelmintic, antiamoebic, antibacterial, antifungal, anti-inflammatory, antioxidant, cytotoxic, hypoglycaemic and immunomodulatory	[88,89]
*Albizia anthelmintica*	Alkaloids, diterpenes, flavonoids, gallic acid, phenolics, saponins and tannins	Analgesic, antibacterial, anti-inflammatory and antioxidant	[90,91]
*Albizia antunesiana*	Coumarins, phenolics and triterpenoids	Anthelmintic and antioxidant	[92]
*Albizia tanganyicensis*	Saponins	Anthelmintic, anticonvulsant, anti-inflammatory, antimicrobial, antioxidant and wound healing	[93,94,95]
*Albizia versicolor*	Glycosides, saponins and triterpenes	Anthelmintic and antifungal	[84,96,97]
*Arachis hypogaea*	Alkaloids, phenolics, phytic acid and saponins	Antioxidant	[98]
*Baikiaea plurijuga*	Alkaloids, anthraquinones, flavonoids, phenolics and tannins	Antibacterial and antioxidant	[99,100,101]
*Bauhinia galpinii*	Fatty acids, flavonoids, phenols, proanthocyanidin, tannins and terpenoids	Antibacterial, antifungal, anti-inflammatory, antioxidant and cytotoxic	[102,103,104,105,106]
*Bauhinia petersiana*	Anthraquinones, alkaloids, cardenolides, flavonoids, saponins, tannins and terpenoids	Antibacterial, antifungal, anti-inflammatory, antioxidative and cytotoxic	[102,104]
*Bobgunnia madagascariensis*	Flavonoids, saponins and tannins	Antibacterial	[107]
*Bolusanthus speciosus*	Alkaloids, flavonoids, phenolics, saponins, tannins and volatile oils	Anti-arthritic, antibacterial, antigonococcal, antimycobacterial, antifungal, anti-HIV, anti-inflammatory and antioxidant	[108]
*Brachystegia boehmii*	Tannins	Antibacterial, anti-inflammatory and antioxidant	[109,110,111]
*Brachystegia spiciformis*	Proanthocyanadin and tannins	* None found	[112,113]
*Burkea africana*	Flavonoids, glycosides, saponins, steroids, tannins and triterpenes	Analgesic, antibacterial, antiviral, anticholinesterase, anti-inflammatory and antioxidant	[68,114,115,116]
*Cajanus cajan*	Coumarins, flavonoids, phenolics and stilbenes	Antioxidant and anti-inflammatory	[117]
*Cassia abbreviata*	Anthocyanins, anthranoids, anthraquinones, polyphenols and tannins	Abortifacient, anti-diabetic, anti-inflammatory, antimicrobial, antiviral, antioxidant and hepatoprotective	[118,119,120]
*Colophospermum mopane*	Alkaloids, coumarins, diterpenes, flavonoids, polyphenols, proanthocyanidins, saponins, sterols and triterpenes	Antibacterial, antiproliferation, antiprotease, antioxidant and cytotoxic	[121]
*Crotalaria laburnifolia*	Alkaloids	Analgesic, anthelmintic and antimicrobial	[122]
*Dalbergia melanoxylon*	Alkaloids, flavonoids, glycosides and tannins	Analgesic, anti-inflammatory, antimicrobial, antiviral, antioxidant and antipyretic	[123,124,125]
*Dalbergia nitidula*	Flavonoids	Antibacterial, antioxidant and cytotoxic	[126,127]
*Dalbergiella nyasae*	Alkaloids, flavonoids, saponins and terpenoids	Antifungal and antibacterial	[128]
*Dichrostachys cinerea*	Flavonoids, phenolics, sterols, tannins and triterpenes	Analgesic, antibacterial, anti-fungal, antiviral, anticonvulsant, anti-inflammatory, antimalarial, antioxidant, hepatoprotective and neuropharmacological	[129,130,131,132]
*Dolichos kilimandscharicus*	Flavonoids and saponins	Antibacterial, anticancer, antiproliferative and cytotoxic	[133,134]
*Elephantorrhiza burkei*	Alkaloids, flavonoids, glycosides, phenolics, saponins, tannins and triterpenoids	Antibacterial, antifungal, anti-HIV, antidiabetic, anti-inflammatory, antioxidant, cytotoxic and mutagenic	[135,136]
*Elephantorrhiza elephantina*	Anthocyanidins, anthraquinones, esters, fatty acids, flavonoids, glycosides, phenolics, saponins, sterols, tannins and triterpenoids	Anthelmintic, antibacterial, antifungal, anti-inflammatory, antinociceptive, antiplasmodial and antioxidant	[137]
*Elephantorrhiza goetzei*	Coumarins, flavonoids, phenolic, saponins, stilbenoids, tannins and triterpenoids	Anthelmintic, antibacterial, antifungal, antiviral, antioxidant and cytotoxic	[138]
*Eriosema englerianum*	Volatile oils	Antibacterial and antifungal	[139]
*Erythrina abyssinica*	Alkaloids, flavonoids and terpenoids	Antibacterial, antifungal, antiviral, antidiabetic, anti-inflammatory, antioxidant, antiplasmodial, antiproliferative and hepatoprotective	[140,141,142,143,144]
*Erythrina livingstoniana*	Flavonoids	Antibacterial and antioxidant	[145,146,147]
*Erythrophleum africanum*	Alkaloids, flavonoids, glycosides, saponins, steroids, tannins and terpenoids	Antibacterial, antifungal, antidote, antioxidant and toxic	[148]
*Erythrophleum suaveolens*	Alkaloids, flavonoids, sterols, stilbenoids and terpenoids	Antibacterial, antifungal, anticancer, anti-inflammatory and antioxidant	[149]
*Flemingia grahamiana*	Alkaloids, flavonoids, glycosides, phenolics, saponins, steroids, tannins and volatile oils	Antibacterial and anticancer	[150,151]
*Indigofera arrecta*	Alkaloids, flavonoids, glycosides, phenols, saponins, tannins and terpenoids	Antibacterial, antiviral and anticancer	[152,153]
*Indigofera astragalina*	Saponins and tannins	Antioxidant and cytotoxic	[154]
*Indigofera hirsuta*	Alkaloids, flavonoids and phenolics	Antidiabetic, anti-inflammatory and antioxidant	[153]
*Indigofera spicata*	Benzofuran, fatty acids, flavonoids, phthalate, rotenoids, saponins, steroids and triterpenes	Anticancer, antidiabetic, antidiarrhoeal, antiplasmodial and cytotoxicity	[153,155]
*Julbernardia globiflora*	Fatty acids, lignin, proanthocyanidins and tannins	Anticancer	[112,156]
*Lessertia frutescens*	Amino acids, flavonoids, pinitol and triterpenes	Analgesic, antibacterial, anticonvulsant, antidiabetic, anti-HIV, anti-inflammatory, antiproliferative, antistress and antithrombotic	[157]
*Leucaena leucocephala*	Coumarins, flavonoids, phytol, sterols and triterpenes	Antimicrobial, diuretic, antiviral, cytotoxic, antioxidant and anti-inflammatory	[158,159,160,161]
*Mucuna coriacea*	None found	Bacterial and antiviral	[162,163]
*Mundulea sericea*	Coumarins, flavonoids, phenolic, saponins, steroids, tannins and volatile oils	Analgesic, antibacterial, antifungal, antioxidant and insecticidal	[164,165]
*Neorautanenia mitis*	Alkaloids, flavonoids, glycosides, saponins and tannins	Antibacterial, antifungal and antinociceptive	[166,167]
*Ormocarpum kirkii*	Coumarins, flavonoids and triterpenoids	Antibacterial, antifungal, antimalarial, antiplasmodial and cytotoxicity	[168]
*Ormocarpum trichocarpum*	Aliphatic hydrocarbons, coumarins, diterpenoids, steroids and triterpenes	Antimicrobial, antiplasmodial, antioxidant and antimutagenic	[169,170,171]
*Peltophorum africanum*	Benzenoids, coumarins, flavonoids, glycosides, phenolics, steroids, tannins and terpenes	Anthelmintic, antibacterial, antifungal, antiviral, anti-inflammatory and antioxidant	[141,162,163,172,173,174]
*Pericopsis angolensis*	Flavonoids, saponins and tannins	Antimicrobial	[107]
*Phaseolus vulgaris*	Alkaloids, anthocyanins, esters, flavonoids, iridoids, lignans, phenolics, saponins, steroids, tannins and terpenoids	Analgesic, antibacterial, antidiabetic, anti-inflammatory, antioxidant and hypocholesterolaemic	[175,176]
*Philenoptera violacea*	Alkaloids, flavonoids, glycosides, steroids, tannins and terpenoids	Anticancer and antioxidant	[177]
*Piliostigma thonningii*	Alkaloids, flavonoids, saponins, tannins, terpenes and volatile oils	Analgesic, anthelminthic, antibacterial, antiviral, antimalarial, anti-inflammatory, antileishmanial, antioxidant, antipyretic and immunomodulatory	[178,179,180,181,182]
*Pseudarthria hookeri*	Flavonoids	Antibacterial and anticancer	[183]
*Pterocarpus angolensis*	Chalcones, deoxybenzoin, fatty acids, phenolics and terpenoids	Antibacterial, antifungal, antiviral, anticancer, anti-inflammatory, antioxidant and wound healing	[184,185,186,187,188]
*Pterocarpus rotundifolius*	Fatty acids	Antiacne, antityrosinase, antioxidant and cytotoxic	[189]
*Pterolobium stellatum*	Saponins, tannins and terpenoids	Antibacterial and antimycobacterial	[190,191]
*Rhynchosia insignis*	Flavonoids	Antibacterial and antifungal	[192,193,194]
*Rhynchosia minima*	Coumarins, flavonoids, steroids, tannins, triterpenes and volatile oils	Antibacterial, antifungal and antioxidant	[47,192,194]
*Rhynchosia resinosa*	Saponins, steroids and terpenoids	Antibacterial, antileishmanial, cytoprotective and cytotoxic	[195,196]
*Schotia brachypetala*	Anthocyanins, flavonoids, glycosides, phenols and tannins	Anti-acne, antibacterial, antimalarial, antioxidant, antityrosinase and cytotoxic	[189,197,198]
*Senegalia ataxacantha*	Alkaloids, coumarins, flavonoids, lignan, phenols, quinone, saponins, steroids, tannins and triterpenoids	Antibacterial, antifungal, antidiabetic, anti-inflammatory, antioxidant, laxative and ulceroprotective	[199]
*Senegalia mellifera*	Flavonoids, glycosides, phenols, saponins, tannins and terpenoids	Antibacterial, antifungal and cytotoxicity	[200,201,202]
*Senegalia nigrescens*	Flavonoids and triterpenoids	Antimicrobial, antioxidant and cytotoxicity	[203]
*Senna didymobotrya*	Alkaloids, flavonoids, phenolics, quinones, saponins, steroids, tannins and terpenoids	Antibacterial	[204,205]
*Senna italica*	Alkaloids, anthocyanins, flavonoids, steroids and tannins	Antibacterial, antifungal, anticancer and antioxidant	[206,207,208]
*Senna occidentalis*	Alkaloids, anthraquinones, anthrones, flavonoids, saponins, sterols and volatile oils	Antibacterial, antifungal, anticancer, antidiabetic, anti-inflammatory, antimutagenic, antiprotozoal and hepatoprotective	[208,209]
*Senna petersiana*	Flavonoids	Antibacterial and cytotoxic	[135]
*Senna singueana*	Alkaloids, anthraquinones, proanthocyanidins, phenols, saponins, sterols, tannins, terpenes and volatile oils	Antimalarial, antinociceptive, antioxidant, hepatoprotective and trypanocidal	[198,208]
*Senna septemtrionalis*	Anthraquinones, benzoic acids, carboxylic acids and flavonoids	Anticonvulsant, anti-inflammatory, diuretic and antinociceptive	[208,210,211]
*Sphenostylis erecta*	Flavonoids and sphenostylins	Antifungal, antioxidant and cytotoxicity	[212]
*Tamarindus indica*	Amino acids, fatty acids and tannins	Antibacterial, antifungal, antiviral, antidiabetic, anti-inflammatory, antinematodal, antioxidant, cytotoxic and molluscicidal	[213]
*Tylosema fassoglense*	Alkaloids, flavonoids, glycosides, phenolics, quinones, saponins, steroids, tannins and terpenoids	Antibacterial and anticancer	[214,215]
*Vachellia karroo*	Flavonoids, phenols, proanthocyanidin, sterols, tannins and terpenoids	Analgesic, antibacterial, antifungal, antiviral, antihelmintic, anti-inflammatory, antimalarial and antioxidant	[216]
*Vachellia nilotica*	Alkaloids, fatty acids, flavonoids and tannins	Inhibition of acetylcholinesterase, anthelmintic, antibacterial, anticancer, antihypertensive, anti-inflammatory, antioxidant and antiplatelet	[217]
*Vachellia rehmanniana*	None found	Anti-inflammatory	[218]
*Vachellia sieberiana*	Flavonoids, glycosides, phenolics, quinones, saponins and tannins	Antibacterial and anticancer	[219,220]
*Vigna unguiculata*	Flavonoids and phenolics	Acetylcholinesterase inhibition, anthelmintic, antibacterial, antifungal, antiviral, antidiabetic, anti-inflammatory, antioxidant, antinociceptive and hypocholesterolaemic	[221,222,223,224,225]
*Xeroderris stuhlmannii*	Alkaloids, flavonoids, phenols, steroids and terpenoids	Antibacterial, antiviral, anticancer, anti-inflammatory, antioxidant and antiproliferative	[226,227,228]

* “No report found” means that no record of the phytochemical or pharmacological properties were found in the literature.

## Data Availability

Not applicable.
